# Effect of the Incorporation of EuCl_3_ as a Fluorescent Probe into Ionic Liquids on Rheological and Electrical Properties for Tribological Applications

**DOI:** 10.1021/acsomega.5c04644

**Published:** 2025-11-06

**Authors:** Jan Blahut, Oldřich Zmeškal, Michal Michalec, Vít Šimara, Petr Svoboda, Rastislav Smolka, Patrik Sokola

**Affiliations:** 1 Faculty of Chemistry, 48274 Brno University of Technology, Purkynova 464/118, Brno 60190, Czech Republic; 2 Faculty of Mechanical Engineering, 48274 Brno University of Technology, Technicka 2896, Brno 60190, Czech Republic

## Abstract

Fluorescence optical microscopy tracking the film thickness as a function of fluorescence intensity under the influence of an external electric field could be a very promising technique. Due to the insufficient fluorescence properties of ionic liquids, the incorporation of fluorescent probes is necessary. This research work investigates the incorporation of europium­(III) chloride (EuCl_3_) as a fluorescent probe into the ionic liquid 1-hexyl-3-methylimidazolium chloride (HMIMCl) and its impact on the rheological and electrical properties of the resulting ionic liquid complex for future tribological use. The study showed that the incorporation of EuCl_3_ imparts fluorescence properties to HMIMCl, characterized by two emission peaks at 590 and 612 nm, which correspond to two specific electronic transitions of the europium ion, namely, ^5^D_0_ → ^7^F_1_ and ^5^D_0_ → ^7^F_2_. Temperature-dependent current–voltage characteristics were measured, which showed increased conductivity correlated with reduced viscosity. Rheological analysis demonstrated that the addition of EuCl_3_ increased the dynamic viscosity of the ionic liquid while remaining within the range of common low viscosity tribological oils. This research highlights the potential of fluorescent ionic liquids in tribological applications and paves the way for the development of smart, environmentally friendly lubricants, which would be able to react to an electric field. The findings contribute valuable insights into the design and optimization of smart fluids that can adapt their properties in response to external stimuli.

## Graphical abstract

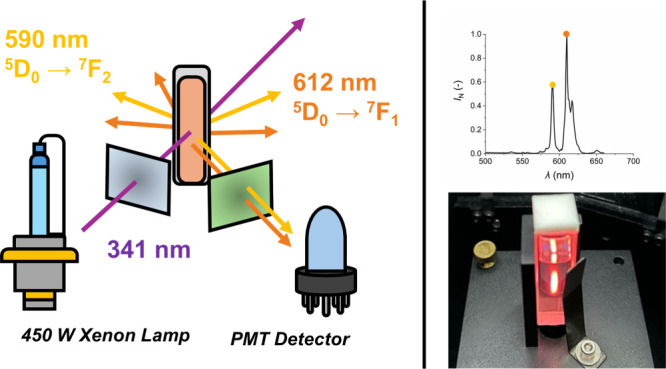

## Introduction

Ionic liquids, first mentioned in the early 20th century,^1,2^ are a promising group of substances with a wide range of potential applications in several scientific fields, such as biomedical applications,^3^ lubricants in tribology,^4^ enhanced lithium batteries,^5^ drug delivery and therapy,^6^ next generation energy devices,^7^ and electrochemical devices,^8^ due to their unique properties, such as high thermal stability, nonflammability,^9,10^ anticorrosion and antimicrobial activities,^11^ low vapor pressure,^12^ high sensitivity to electric fields,^13^ and extensive liquid range.^14^ These properties together with their unusual solvation properties and highly tunable properties by cation and anion variation and combination promote them as “green” solvents.^10^

Ionic liquids are mostly composed of a larger organic cation with very low symmetry and a smaller inorganic or organic anion.^10^ Among the cations, representatives of five- and six-membered heterocyclic compounds appear, such as imidazolium,^10,15^ pyrrolidinium,^16^ and pyridinium and pyrylium salts,^17^ as well as ammonium,^4^ phosphonium,^18^ and sulfonium salts with various substituents.^10^ Anions are mainly formed by halides,^19^ nitrates,^20^ hexafluorophosphates and phosphates,^21,22^ borates and tetrafluoroborates,^23,24^ acetates,^25^ or derivatives of more complex substances such as bis­(trifluoromethanesulfonyl)­imide.^26,27^

One of the main fields that has shown great interest in ionic liquids in recent years is tribology. Ionic liquids form the basis of several functional lubricants due to their useful properties.^4,28,29^ The main role of lubricants is to minimize wear of contact bodies and reduce friction.^30,31^ Due to their widespread use, there is a growing demand for “green” alternatives to currently used synthetic oils.^32^ These oils are more than satisfactory in terms of lubricating properties; however, they carry several unsolved problems that need to be addressed, namely, the contact starvation effect that occurs during the elastohydrodynamic lubrication regime, dielectric breakdown, or electric discharge. During the elastohydrodynamic lubrication regime, the film between the two interfaces is influenced by lubricant viscosity, sliding speed, and equipment load. If a sufficient supply of lubricant is not ensured, the mentioned effect of contact starvation may occur, which causes excessive overheating of the device, increased material wear, contamination, or even lead to a failure of a machine.^33^ More negative effects for synthetic oils are brought about by the modern trends of electrification, such as dielectric breakdown or electric discharge. The risk of these effects is significantly higher when nonconductive lubricants are used. In an electrified contact lubricated with an insulating lubricant, electric discharge in the form of arcing would happen when the voltage exceeds the breakdown potential of the lubricant film, leading to potentially catastrophic surface damage.^34,35^

Ionic liquids seem like ideal candidates to solve these current tribological problems at this point. Ionic liquids belong to the group of so-called smart materials, or smart fluids, which can change their rheological and tribological properties by external stimuli, such as light, heat, or especially magnetic and electric fields.^36^ It is the effect of the last-mentioned electric field on the properties of the ionic liquids that is currently the target of intensive research. Ionic liquids that respond to excitation by an electric field are called smart electrorheological fluids,^37^ and understanding and describing the mechanism of the behavior of ionic liquids under this effect may in the future lead to the complete replacement of synthetic oils by these smart fluids.

Several instrumental methods and devices are currently used to study the tribological properties of lubricants, such as the optical tribometer for film thickness evaluation and the Mini-Traction Machine for friction measurements.^38^ There is a tendency to invent new methods or upgrade already existing ones because such devices are not designed to incorporate external electrical circuits that are necessary for influencing the properties of electrorheological ionic liquids. Both optical tribometers and Mini-Traction Machines have been used in the past to investigate smart ionic liquids; however, the devices were damaged, for example, due to electrochemical reactions that occurred as a result of the combination of the use of conductive ionic liquids and an electric field, as mentioned by Michalec et al.^38^ At the same time, it is not possible to observe ionic liquid excitation mechanisms in contact with available technology. A very promising method could be fluorescence microscopy monitoring the film thickness influenced by an external electric field as a function of fluorescence intensity.^39^

Although some ionic liquids exhibit fluorescence properties, the radiation intensity is usually very low.^40^ Therefore, it is necessary to use fluorescent probes that impart these properties to ionic liquids. Lanthanide salts, which are used as fluorescent probes,^41–43^ offer several important properties that can be used to influence the resulting fluorescence of ionic liquids. By combining different ionic liquids and different lanthanides, the wavelength of the absorbed and emitted radiation, the lifetime of the excited states, etc. can be varied. In principle, there are two approaches to the synthesis of fluorescently activated ionic liquids. The first method is to simply dissolve the lanthanide salt of cation Ln^2+^ or Ln^3+^ in a suitable ionic liquid. This method can only achieve very low concentrations of dissolved probes due to the lower solubility of lanthanide salts in ionic liquids. Solvation phenomena in ionic liquids are not as straightforward as in classical solvents. Dissolution does not mean dissociation and solvation of the metal cation only by the anions of the ionic liquids. The overall solubility is very strongly dependent on the choice of ions of the ionic liquids and the dissolved salt.^44^ To achieve better stability of the resulting solutions and higher reproducibility, sufficient characterization and possible purification of the ionic liquids used are required.^45^ In the case of using chloride salts of lanthanides, chloride anions play a significant role because they cause the occurrence of important nonbonding interactions in the form of hydrogen bonds, which significantly affect the solubility of the lanthanide salt in ionic liquid.^46^ However, if the anion of the ionic liquid has coordination properties, a second method of incorporation can be applied, which is the formation of lanthanide based ionic liquid (Ln-IL) complexes, where the lanthanide cation appears as the central atom and the ionic liquid molecules act as ligands.^43^ However, Ln-ILs differ from other complexes in certain properties, such as weakly coordinating anions, which leads to different solubilities compared to conventional solvents. This can result in coordination compounds with low coordination numbers and enhanced reactivity in organic reactions.^47^ Nevertheless, high quantum yields can be achieved even with weakly coordinating ionic liquids.^48^ Some Ln-IL complexes are relatively unstable and thus require additional stabilization with neutral coligands.^49^ Some Ln-ILs have very remarkable properties, such as large pseudo-Stokes shifts or long emission lifetimes. For these reasons, this approach is preferred in many applications.^50^

This study focuses on the preparation of 1-hexyl-3-methylimidazolium chloride ionic liquid (HMIMCl) enriched with fluorescence properties and the effect of probe incorporation europium­(III) chloride (EuCl_3_) on the resulting tribological, rheological, and electrical properties of the resulting ionic liquids. The combination of HMIMCl and EuCl_3_ was chosen as an ideal option for the preparation of an ionic liquid complex exhibiting fluorescence properties. The reason for investigating these properties is the high potential for using ionic liquids with fluorescence properties in instrumental methods that can enhance the supplementation of existing methods.

## Materials and Methods

1-Hexyl-3-methylimidazolium chloride (≥97.0%) and anhydrous EuCl_3_ (99.99%) for the synthesis of Ln-IL complex were purchased from Sigma-Aldrich. Chemicals were stored in accordance with the safety data sheets.

### Sample Preparation

#### Synthesis

The reaction principle of the synthesis of Ln-ILs is based on the dissolution of Ln salts in coordinating ionic liquids. The reaction equation for the synthesis of Eu-HMIMCl is shown in Figure 1. The solution of EuCl_3_ in HMIMCl (2 wt %) was prepared by dissolving EuCl_3_ in HMIMCl at 90 °C with stirring (400 rpm) for 2 h, which resulted in the formation of an appropriate amount of Eu-HMIMCl complex. After the mixture was stirred, water was added to the complex to reduce the viscosity to form an aqueous solution of Eu-HMIMCl. The water ratio (0.56) was chosen in advance so that the viscosity of the samples being determined was as close as possible to the viscosities of tribological oils.^51^ The whole procedure took place within a glovebox under an argon atmosphere to protect the prepared complex from additional moisture.

**Figure 1:**
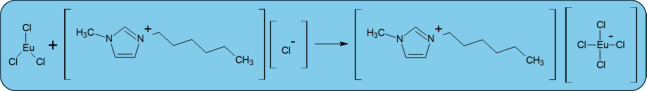
Eu-HMIMCl complex formation process.

### Materials Characterization

#### Fluorescence

UV–vis absorption spectra were measured using a Varian Cary UV–vis 50 spectrophotometer. Samples were diluted with solvent to ensure that the absorbance above 340 nm did not exceed 0.1 to avoid reabsorption.

To verify the fluorescence properties of HMIMCl, emission spectra were recorded at four excitation wavelengths: 300, 350, 450, and 500 nm. Steady-state photoluminescence spectra were collected using a FluoroLog-3 (HORIBA) spectrofluorometer over the 325–800 nm range, with a resolution of 1 nm. The instrument was equipped with double-grating monochromators, a photomultiplier tube (PMT) detector, and 450 W xenon lamp excitation source, with measurement conducted at 20 °C. The excitation and emission slit widths were adjusted to prevent detector saturation, ensuring that the PMT signal did not exceed 10^6^ counts per second due to detector saturation. The acquisition time for a single measurement was kept in the range of 2–4 min, with the resulting spectrum being the average of three consecutive measurements. The absolute fluorescence quantum yield (Φ_FL_) in solution was determined using the calibrated integrated sphere Quanta-φ (HORIBA).

The excitation and emission spectra of the Eu-HMIMCl complex were measured using the same equipment and under the same conditions as the emission spectra of pure HMIMCl. Emission spectra of the complex were measured for two wavelengths (341 and 464 nm), belonging to the two highest peaks in the obtained excitation spectrum.

#### Dynamic Viscosity

Viscosity measurements were carried out by using a HAAKE RotoVisco 1 instrument equipped with a temperature-controlled measuring chamber in the double gap Couette geometry configuration. Four individual measurements were carried out for each temperature, each within a shear rate range from 100 to 10 s^–1^ in the six steps, each step consisting of a pre shear and a 5 s recorded part. The dynamic viscosity for each temperature was determined by linear regression from all stress–strain dependence data. The correlation coefficient exceeded 0.99 in all cases. The temperature of the tempering oil surrounding the measuring cylinder was monitored and recorded for each measurement with a maximum standard deviation of 0.03 °C.

#### Current–Voltage Characteristics

The electrical properties of the pure ionic liquid and the ionic liquid with the incorporated fluorescent probe in the dark and under a xenon lamp were determined. To verify that the ionic liquid with incorporated europium atoms retained its original electrical properties, we measured the current–voltage (*I*–*V*) characteristics.

The *I*–*V* characteristics were measured in several cycles always in the voltage range from −10 to 10 V. For each additional cycle, the temperature was increased by heating the substrate with a constant power with a step of 1.5 °C in the range of 25 to 100 °C. During one cycle, which lasted for 60 min, the temperature was maintained by a thermostatic bath based on the circulation pump principle with microprocessor technology and an integrated cooling system LAUDA ECO RE 415 S. A Keithley 2410 High Voltage SourceMeter (110 V, 1 A, 20 W) was used for this measurement as the electric field source.

## Results and Discussion

### Fluorescence Properties

Figure 2 shows how the absorbance of the sample changed with 10 and 20× solvent dilution. The 10× diluted solution was subsequently used to determine Φ_FL_ because the absorbance value for the excitation wavelengths of 341 and 464 nm did not exceed 0.1.

**Figure 2:**
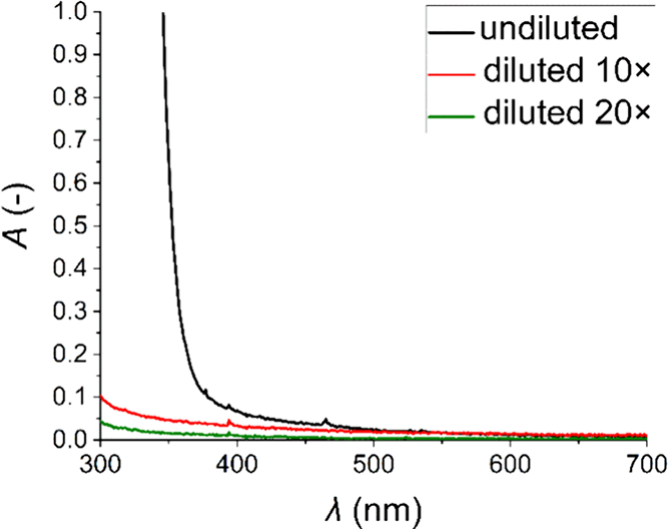
Absorption spectra of the Eu-HMIMCl solution.

The absolute quantum yield of this prepared Eu-HMIMCl solution reached very low values, namely, <1%, while other ionic liquids form complexes with europium atoms with much higher values from 32% to 64%.^52–54^ The choice of an anion plays a key role here, with Tf_2_N^–^ and PF_6_^–^ anions being preferred for their greater molecular volume and weaker cooperative ability.^54^ HMIMCl appears to be a challenging choice for evaluating the quantum yield, as discussed in this paragraph, moving in the direction of other ionic liquids and luminescent probes or possibly helping with the addition of different ligands such as β-diketonates.^53^

The measured emission spectra of pure HMIMCl showed the presence of emitted radiation (Figure 3). It is also evident that the highest radiation peak moves toward higher wavelengths with increasing excitation wavelength of the source. This behavior, called the red-edge effect, is characteristic of ionic liquids with an imidazolium cation and is relatively uncommon.^40^

**Figure 3:**
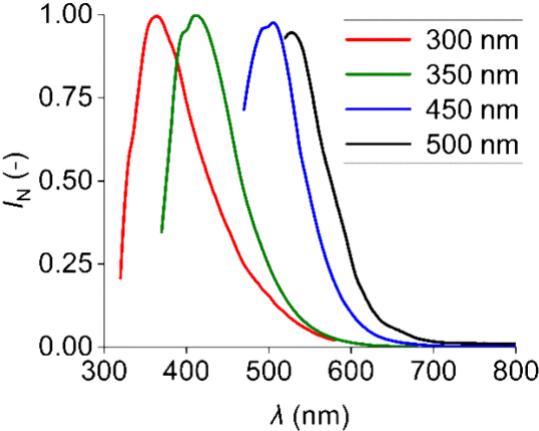
Normalized emission spectra of pure HMIMCl.

This behavior has been predicted and explained in the literature^55,56^ by the existence of heterogeneity in the ionic liquids. The long trapping time of molecules in quasi-static local solvent cages results in site-specific absorption and emission characteristics, causing subsets of fluorescent molecules to absorb and emit at different frequencies based on their local environment, a phenomenon not observed in typical solvents such as methanol.

The normalized excitation spectrum (Figure 4) of a prepared solution of Eu-HMIMCl complex in water shows the two highest peaks belonging to 341 and 464 nm. These two wavelengths were selected to measure the emission spectra of the Eu-HMIMCl complex.

**Figure 4:**
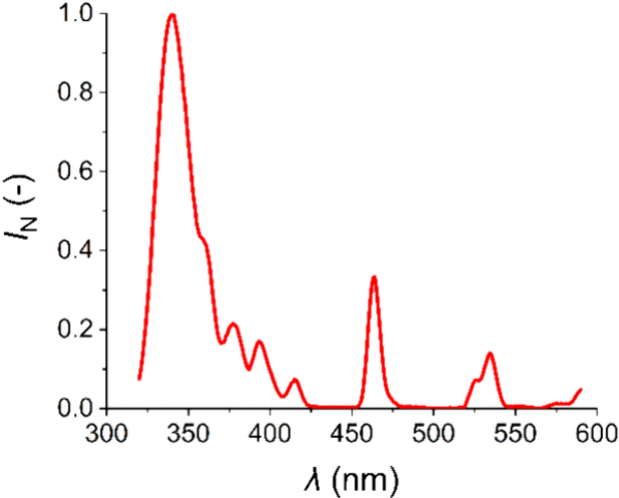
Normalized excitation spectrum of a solution of Eu-HMIMCl complex in water.

For both excitation wavelengths, the normalized emission spectra of the Eu-HMIMCl complex (Figure 5a, b) are very similar. Two characteristic peaks for the Eu^3+^ ions can be observed in both spectra. The first peak belongs to the ^5^D_0_ → ^7^F_1_ transition, which lays around 590 nm in the orange area. The second and the most important characteristic peak is located around 612 nm in the red area of the spectrum and belongs to the ^5^D_0_ → ^7^F_2_ transition.^57^ This transition is most intense in combination with ionic liquids.^58^ The luminescence is visible to the naked eye due to these two transitions (Figure 6).

**Figure 5:**
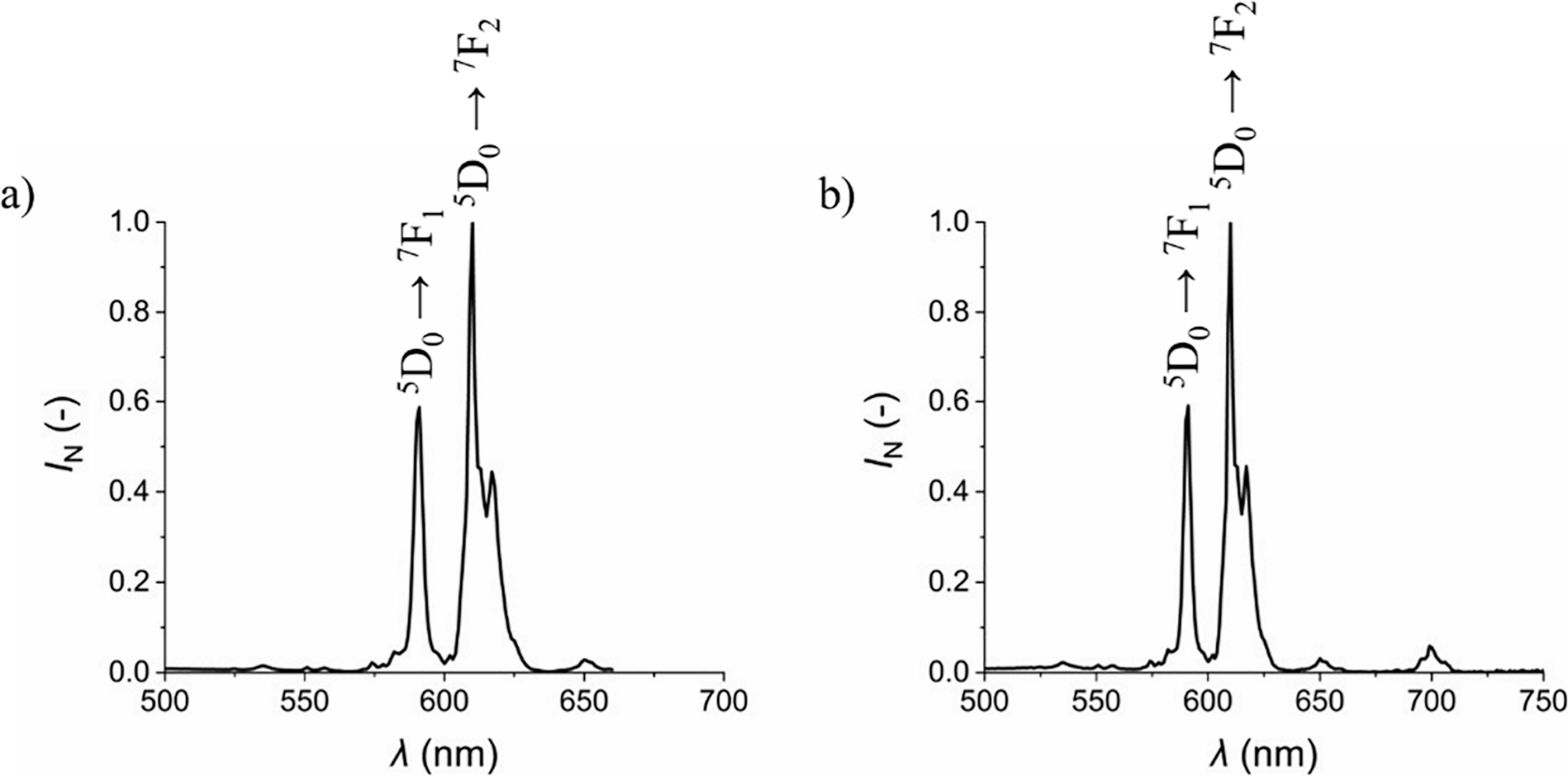
Normalized emission spectra of the Eu-HMIMCl complex for 341 (a) and 464 nm (b).

**Figure 6:**
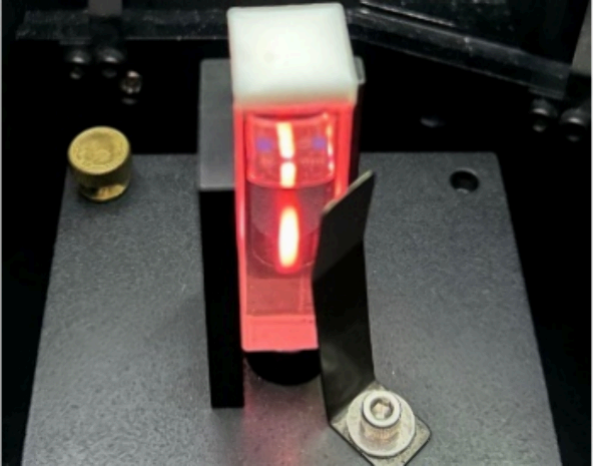
Fluorescence of the Eu-HMIMCl complex characteristic of the ^5^D_0_ → ^7^F_1_ and ^5^D_0_ → ^7^F_2_ transitions.

The position of the characteristic peaks of Eu^3+^ can vary depending on many factors; for example, the presence of coordinating groups such as phosphoryl in ionic liquids can lead to a shift in the emission peaks due to changes in the local symmetry and coordination environment around the Eu^3^^+^ ion.^58^

At the same time, the emission spectra of the anhydrous solution of the Eu-HMIMCl complex and the aqueous solution of HMIMCl were measured at an excitation wavelength of 341 nm. All of the mentioned spectra are shown in Figure 7. Adding a small amount of water to the ionic liquid can cause a shift to the red spectrum,^59^ which is not completely noticeable in our case. The emission spectrum of the aqueous solution of HMIMCl consists of a broad peak, the coordinates of which correlate with the peaks in Figure 3. When comparing the aqueous and anhydrous solutions of the Eu-HMIMCl complex, this peak is the only difference in both spectra; however, in terms of intensity, it is almost insignificant compared to other more intense peaks characteristic of europium ions. As for the solution of EuCl_3_ in water, the spectra of this solution have been well-known for a long time. In these solutions, as in the previous cases, the two transitions ^5^D_0_ → ^7^F_1_ and ^5^D_0_ → ^7^F_2_ dominate, but others also occur, e.g., ^5^D_0_ → ^7^F_0_, ^5^D_0_ → ^7^F_3_, or ^5^D_0_ → ^7^F_4_.^60^

**Figure 7:**
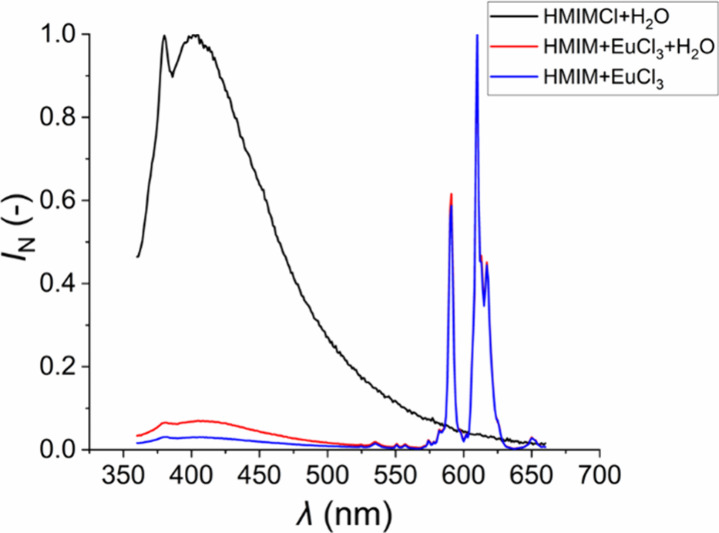
Emission spectra of an aqueous solution of the ionic liquid, an anhydrous solution of the Eu-HMIMCl complex, and an aqueous solution of the Eu-HMIMCl complex.

The Eu-HMIMCl complex was also excited by other wavelengths corresponding to other visible peaks in the measured excitation spectrum (Figure 4), e.g., 361, 379, 393, 416, 525, 535, and 591 nm. An interesting fact is evident from Figure 8. The observed light from the sample changes with increasing wavelength of the excitation source. This phenomenon occurs due to scattering of radiation by relatively large molecules of the ionic liquid and its complex. At higher excitation wavelengths, i.e., wavelengths that do not correspond to the excitation maximum of the molecules in the sample, the fluorescence of the sample is overwhelmed by the scattered radiation from the source.

**Figure 8:**
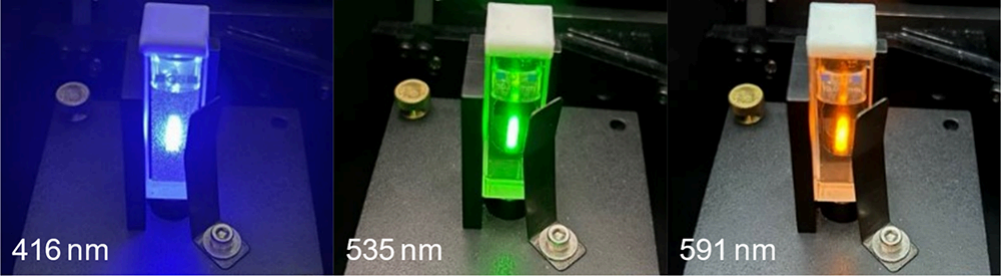
Change in sample color due to scattering and increasing wavelength of the excitation source.

### Rheological Properties

The measured dependences of the dynamic viscosity of HMIMCl and Eu-HMIMCl on temperature (Figure 9) show an exponential decrease in dynamic viscosity with temperature, and in fact, most ionic liquids exhibit this very behavior.^61^ The decrease in dynamic viscosity is caused by an increase in thermal energy in the system, which disrupts intermolecular interactions and thus facilitates the movement of ions.^62^ The dynamic viscosities of both samples for given concentrations in the range of 20 to 40 °C range from 120 to 20 mPa s, which corresponds in order to the dynamic viscosities of commonly used tribological oils, such as polyalphaolefin based oils.^63,64^

**Figure 9:**
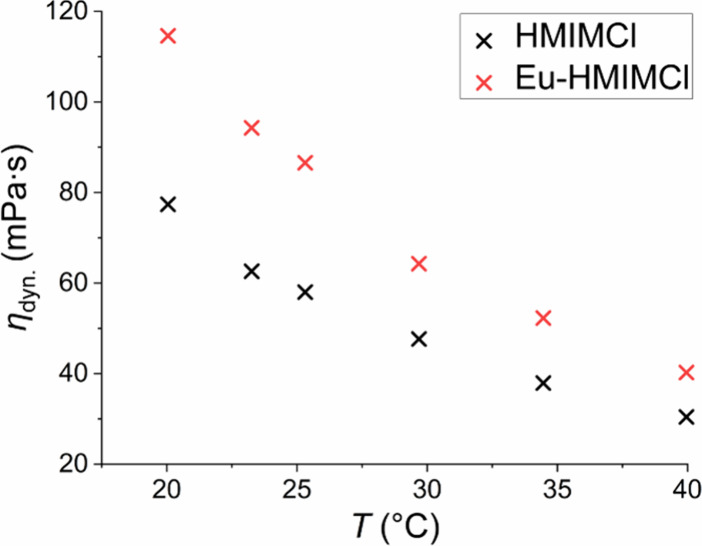
Temperature dependence of the dynamic viscosities of HMIMCl and Eu-HMIMCl.

Another fact can be noted from Figure 9, namely, that after incorporating the fluorescent probe into the ionic liquid, its viscosity visibly increased. This phenomenon is not intuitive at first because the general rule is that as the size of the anion in an ionic liquid increases, the viscosity decreases. This is caused by poorer particle mobility or the formation of larger and less mobile aggregates.^65,66^ However, the size of the ions is not the only factor that affects the viscosity. Viscosity is also affected by, for example, van der Waals forces, electrostatic interactions, etc., which have a greater influence on viscosity.^67^ The addition of EuCl_3_ to the ionic liquid increased the ion concentration, which caused a greater number of electrostatic interactions and increased the overall viscosity of the system.

### Electric Properties

Each of the measured *I*–*V* characteristics for all three samples was evaluated separately in the following manner. First, the values of the short-circuit current (*I*_SC_) and open-circuit voltage (*U*_OC_) as intercepts with the *x* and *y* axes, two important parameters of electrical behavior of ionic liquids, were found. The short-circuit current value was then subtracted from the measured characteristics to shift the entire curve and pass through the origin (Figure 10).

**Figure 10:**
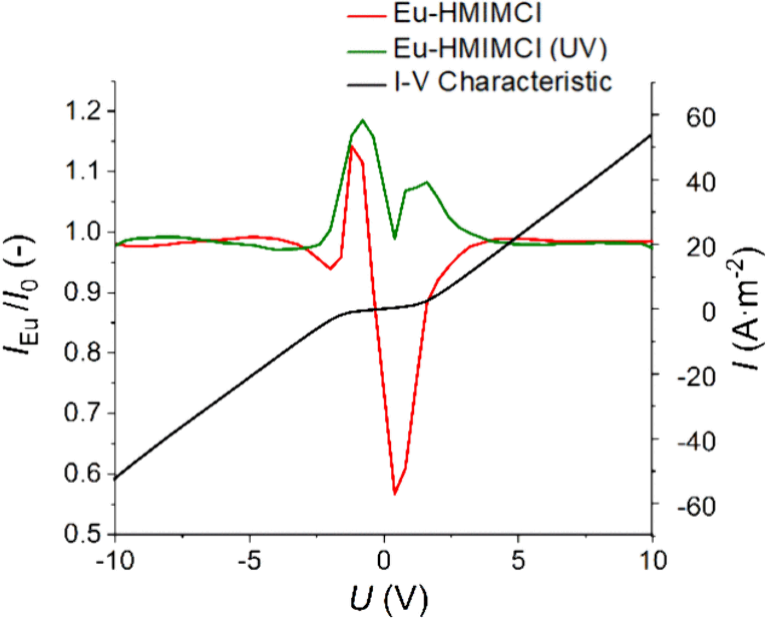
*I*–*V* characteristic after deduction of the value of the *I*_SC_ current at 25 °C.

The parameter *U*_OC_ is the voltage measured across the terminals of a device when no current flows. The *U*_OC_ provides important information on electrochemical devices such as solar cells, fuel cells, and batteries. In most cases, a higher *U*_OC_ indicates a higher energy reserve in the system and thus a higher efficiency of the device.^68,69^

At first glance, the *U*_OC_ values appear to be independent of temperature; however, over the entire temperature range, their decrease in the order of 10^–1^ V is visible for all samples. This behavior is typical for most electrochemical processes and devices.^70,71^ All *U*_OC_ voltage values are summarized in Table 1.

**Table 1:** Comparison of the Initial and Final *U*_OC_ Voltages for Ionic Liquids

sample	initial voltage *U*_OC_ (V)	final voltage *U*_OC_ (V)
HMIMCl	1.1	1.1
Eu-HMIMCl	1.2	1.1
Eu-HMIMCl under UV	1.1	0.9

For HMIMCl, the *U*_OC_ values were around 1.1 V with a very low decrease. After the incorporation of europium cations, the *U*_OC_ started at 1.2 V and decreased to 1.1 V during the increase in temperature, indicating that the internal energy of the system remained almost unchanged. When Eu-HMIMCl was exposed to UV radiation, the *U*_OC_ value was around 1.1 V and subsequently decreased to 0.9 V. In this case, a slight decrease in the energy of the system can be observed compared to the original two samples since exposing the sample to UV radiation results in the generation of new charge carriers. The ability of UV radiation to generate new charge carriers, such as ions or electrons, depends on several factors and properties, especially the viscosity of the ionic liquid or mutual interactions between its ions. Higher energy sources, such as UV radiation, are then capable of generating so-called solvated electrons under certain circumstances, most often through photoexcitation phenomena or photolysis of halide ions.^72,73^ Another way that UV radiation can increase the concentration of charge carriers is through photodissociation of ion pairs, which leads to the separation of cations and anions.^74^

*I*_SC_ values are important for modeling ion transport systems. In such analysis, electrical components such as resistors or capacitors in combinations of series and parallel connections are used to represent ion channels. This approach allows describing the energy distribution in the systems under study.^75^

Since the values of the currents flowing through ionic liquid are almost identical, the *I*–*V* characteristics are almost indistinguishable at first glance at a given temperature. For this reason, the ratio between the current flowing through HMIMCl after the incorporation of europium and the current of pure HMIMCl is shown in Figure 10. This ratio represents how many times the current increased or decreased after incorporation of the fluorescent probe. For negative voltage polarity in the range of approximately −2 to 0 V, the current increases by 15 to 20% for the Eu-HMIMCl sample both without (red curve) and under UV radiation (green curve). For lower voltages, the currents are almost unchanged. The increase in current through Eu-HMIMCl is expected because additional ions in the form of EuCl_3_ were added to the solution, which means an increase in charge carriers. When the Eu-HMIMCl sample is exposed to UV radiation, additional charge carriers are generated, and the current increases even further. For positive voltages, the situation is the opposite. In the range from 0 to 2 V, the current decreases for both samples since the charge is carried by slower ions in the opposite direction of the electric field. Once the sample was exposed to UV radiation, the decrease was compensated for by the charges generated by the radiation.

Three regions of the *I*–*V* characteristics are visible in Figure 10 (black curve). One is for voltages around the origin, and the other two are for higher and lower voltages, respectively. The first mentioned region is the so-called linear ohmic region, which is characterized by behavior according to Ohm’s law.^77^ If the *I*–*V* characteristic shows deviations from Ohm’s law, in the case of the following region, the increase in current is steeper. Here, the occurrence of the so-called superlinear region is obvious, which is caused by the occurrence of space charge.^78^ This region then very quickly passes into another linear ohmic region, only with a different resistance. For tribological applications, it is the ohmic regions that are of the greatest importance.

As can be seen in Figure 11, it is clear that both current *I*_SC_ conductivity of the ohmic region *G*_Ω_ initially increases sharply with temperature and then saturates. However, at a certain temperature, around 75 °C, both parameters slightly decrease and then remain almost constant. This behavior can be observed for HMIMCl and Eu-HMIMCl samples, but not for Eu-HMIMCl under UV irradiation. This sample shows a slower increase in the values of both parameters, and therefore, in the given temperature range, complete saturation has not yet occurred, i.e., it can be assumed that a decrease in conductivity would occur only at higher temperatures. This behavior has been previously described for several ionic liquids including HMIMCl. At higher temperatures, there is a higher probability of spontaneous ion pair formation, leading to a decrease in the concentration of charge carriers, which will negatively affect the conductivity of the liquid.^76^ The increase in conductivity and other determined electrical parameters are related to a higher ion mobility and decrease in the dynamic viscosity of the samples (Figure 9). Both higher energy and lower dynamic viscosity mean easier movement of ions through the medium and thus easier charge transfer. The temperature dependence of conductivity correlates exactly with the measured temperature dependence of dynamic viscosity. As mentioned above, there is initially a sharp decrease in viscosity and subsequent saturation, which corresponds to a sharp increase and subsequent saturation in conductivity. This behavior is characteristic for most of the ionic liquids.^61,79^

**Figure 11:**
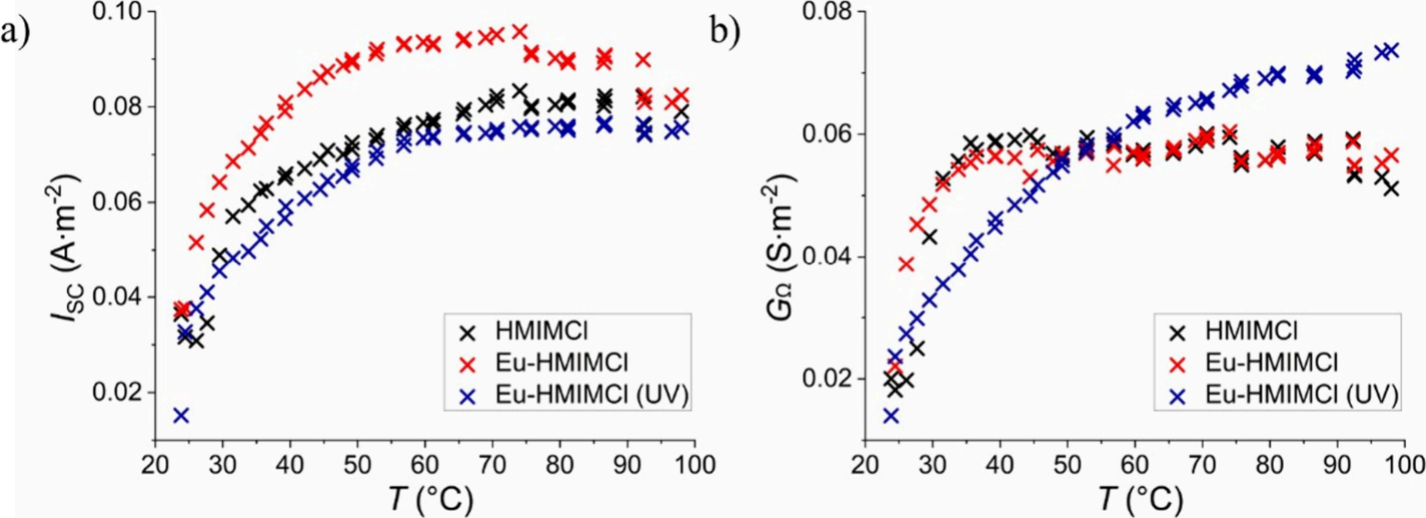
Temperature dependence of short-circuit current (a) and conductivity (b).

## Conclusions

This study addresses the incorporation of EuCl_3_ as a fluorescent probe into ionic liquids and its influence on the rheological and electrical properties for tribological applications. The study provided decent insight into rheological and electrical properties relevant for future applications and utilization.

The incorporation of EuCl_3_ as a fluorescent probe led to the appearance of fluorescence properties in the investigated ionic liquid HMIMCl. The measured emission spectra were characterized by two crucial peaks at 590 and 612 nm belonging to the ^5^D_0_ → ^7^F_1_ and ^5^D_0_ → ^7^F_2_ transitions. At the same time, a rare phenomenon, the so-called red-edge effect typical of ionic liquids, was observed in pure HMIMCl. The last phenomenon that was addressed in the part focused on fluorescence was the visual colour change of the Eu-HMIMCl solution caused by radiation scattering on ionic liquid molecules. The incorporation of europium ions into ionic liquids led to an increase in the dynamic viscosity of the ionic liquid. The values of dynamic viscosity were still within the range of values corresponding to those of the low-viscosity tribological oils used. Not only *I*–*V* characteristics were measured as a function of temperature, which will be used in the future for further characterization of ionic liquids, such as mobility and concentration of charge carriers, but also important electrical parameters of HMIMCl were determined from these characteristics, such as conductivity, short-circuit current, and open-circuit voltage. After incorporation of the fluorescent probe, an increase in the most important of the mentioned parameters, especially the conductivity, was observed. With decreasing viscosity, the conductivity increased, which is a phenomenon observed for most ionic liquids.

Overall, this work opens new possibilities for future study and use of ionic liquids from the perspective of tribological applications. A strong correlation between the electrical and rheological behaviors of ionic liquids was found and experimentally confirmed. The results of this research contribute to the development of more effective and energy-efficient lubricants.

## Abbreviations

EuCl_3_: europium­(III) chloride
HMIMCl: 1-hexyl-3-methylimidazolium chloride
ISC: short-current current
*I*–*V*: current–voltage
Ln-ILs: lanthanide based ionic liquids
*U*_OC_: open-circuit voltage
